# Espindolol for the treatment and prevention of cachexia in patients with stage III/IV non‐small cell lung cancer or colorectal cancer: a randomized, double‐blind, placebo‐controlled, international multicentre phase II study (the ACT‐ONE trial)

**DOI:** 10.1002/jcsm.12126

**Published:** 2016-07-01

**Authors:** Andrew J. Stewart Coats, Gwo Fuang Ho, Kumar Prabhash, Stephan von Haehling, Julia Tilson, Richard Brown, John Beadle, Stefan D. Anker, for and on behalf of the ACT‐ONE study group

**Affiliations:** ^1^ Monash UniversityMelbourneAustralia; ^2^ Warwick UniversityCoventryUK; ^3^ Universiti Malaya MedicalKuala LumpurMalaysia; ^4^ Tata Memorial HospitalMumbaiIndia; ^5^ Division of Innovative Clinical Trials, Department of CardiologyUniversity Medical Center Göttingen (UMG)GöttingenGermany; ^6^ PsiOxus Therapeutics LimitedAbingdonUK

**Keywords:** Cancer, Cachexia, Randomized controlled study, Espindolol

## Abstract

**Background:**

Cancer cachexia is a major cause of morbidity and mortality with no widely approved treatment.

**Methods:**

The ACT‐ONE trial is a randomized, double‐blind, parallel group, placebo‐controlled, phase II multicentre trial in patients (25‐80 years) with stages III or IV colorectal cancer or non‐small cell lung cancer‐related cachexia that tested two doses of espindolol (a novel non‐selective β blocker with central 5‐HT1a and partial β2 receptor agonist effects). The primary endpoint was the difference in the rate of weight change over 16 weeks (linear mixed‐effect model for repeated measures) between high‐dose espindolol and placebo.

**Results:**

Eighty‐seven patients were randomized centrally in blocks in a ratio 3:2:1 [42 high dose, 10 mg twice daily (bd):31 placebo:14 low dose, 2.5 mg bd]. High‐dose espindolol produced a statistically and clinically significant weight gain (+0.54 kg/4 weeks, 95% CI 0.38–0.70) compared with a weight loss on placebo (−0.21 kg/4 weeks, 95% CI ‐0.37–0.05); *P* < 0.0001. High‐dose espindolol produced a statistically significant increase in lean body mass, whilst changes in fat mass were neutral. Hand grip strength significantly (high dose −1.15 ± 0.7 kg, placebo −3.51 ± 0.8 kg change per 4 weeks; *P* = 0.0134), stair climbing power, and 6‐min walk test non‐significantly were all directionally in favour of high‐dose espindolol. There were no clinically significant differences in safety signals or survival between treatment groups, although a numerical excess of dyspnoea was seen with high‐dose espindolol (19.1%) compared with placebo (3.2%).

**Conclusions:**

This positive trial showed that espindolol 10 mg bd significantly reversed weight loss, improved fat free mass, and maintained fat mass in advanced colorectal cancer and non‐small cell lung cancer‐related cachexia. This was associated with a significant improvement in handgrip strength, supporting the further investigation of 10 mg bd espindolol for the treatment of cancer cachexia. Although not powered to look at dose response, most treatment effects for low dose lay between high dose and placebo, suggesting that there may be a dose response in the effects of espindolol.

## Research in context

### Evidence before this study

No widely approved therapeutic agent exists for the treatment or prevention of cancer‐related cachexia. The authors searched the Cochrane Central Register of Controlled Trials, MEDLINE, and EMBASE in June 2015 to identify eligible randomized controlled trials comparing any therapeutic agent with placebo or usual care. Search terms included cancer, cancer‐related cachexia, weight loss, and randomized controlled trials and were restricted to trials recruiting human patients but were not restricted by language. The most convincing published study prior to this report was a phase II study of Enobosarm (GTx‐024; GTx, Memphis, TN, USA), a selective androgen receptor modulator that showed a significant increase in total lean body mass with enobosarm (enobosarm 1 mg: median 1.5 kg increase, range −2.1 to 12.6; *P* = 0.0012; enodosarm 3 mg: median 1.0 kg increase, −4.8 to 11.5; *P* = 0.046) but not in the placebo group (median 0.02 kg, range −5.8 to 6.7; *P* = 0.88). Two phase III trials of Enobosarm in cancer‐related cachexia showed inconsistent results, however. The co‐primary endpoints in both studies were a responder analysis in lean body mass and stair climb power. In one study (514 study), LBM was improved, whereas stair climb power was not. In the second trial (505), neither was improved. These trials have only been presented at conferences and not yet in a peer‐reviewed publication. Anamorelin, an oral ghrelin mimetic, has been tested in several trials. In a cross‐over study in 16 patients with cancer‐related cachexia, anamorelin 50 mg/day over 3 days significantly increased body weight compared with placebo (0.77 kg vs. −0.33 kg), and appetite was reported as being increased. In another set of studies investigating anamorelin for patients with cancer cachexia over 12 weeks, lean body mass decreased by 0.2 kg in patients on placebo, whereas it increased by 1.9 kg in patients on anamorelin [treatment effect 2.09 kg (CI: 0.94–3.25]; *P* = 0.0006). The treatment was also associated with increased non‐dominant hand grip strength (treatment effect 2.59 kg; *P* < 0.02). In the paired phase III trials (Romana 1 and 2), anamorelin improved only one of the two co‐primary endpoints (lean body mass but not hand grip strength) in patients with cancer‐related cachexia (data only in abstract form as yet). L‐Carnitine supplementation has also shown activity in one study of 72 patients with advanced pancreatic cancer and weight loss, body mass index increasing by 3.4 ± 1.4% with L‐Carnitine and decreasing (−1.5 ± 1.4%) in the placebo group; *P* < 0.05. There have been fewer trials employing an anticatabolic approach. Three small studies of TNF‐α inhibitors etanercept, infliximab, and thalidomide failed to show benefits. In an earlier and now abandoned clinical trial programme, the angiotensin‐converting enzyme inhibitor imidapril had been studied in 200 patients with one of three cancer types, and improvement in body weight was reported in two (colorectal cancer and non‐small cell lung cancer) but not in the third type studied (pancreatic cancer) nor in the pre‐specified analysis of all three cancer types taken together.

### Added value of this study

This is the first phase II trial of a combined anabolic and anticatabolic therapy that shows significant effects on fat‐free mass, and the largest change in body weight, combined with a positive effect on a relevant functional measure, hand grip strength, encouraging the agent to be tested in a phase III trial in cancer‐related cachexia.

### Implications of all the available evidence

The multiaction beta‐blocker espindolol has shown the largest body weight gain effect in cancer cachexia in phase II trials to date.

## Introduction

Cachexia (from the Greek: kakos meaning bad and hexis meaning condition) is a wasting disease, associated with significant morbidity and mortality, accompanying a wide range of serious chronic illnesses. It has been defined as weight loss, associated with a chronic underlying disease, of at least 5% in 12 months or less.^1^ Cachexia is commonly associated with fatigue, loss of muscle strength, and fat tissue loss associated with a range of immune, neurohormonal, metabolic, and biochemical abnormalities. It is characteristically associated with negative protein loss, reduced food intake, and abnormal metabolism. Cancer cachexia occurs in a third or more of all patients with cancer and has been estimated to be the direct cause of death in up to 20% of all cancer‐related deaths.^2^ There is currently no widely approved therapeutic agent for treating cancer cachexia. Colorectal cancer (CRC) and non‐small cell lung cancer (NSCLC) have relatively high incidences of cachexia, approximately 50%.^2^

Through its combined pharmacological actions, espindolol (the s‐isomer of the better known pindolol^3^) has a multifunctional effect upon three potential pharmacological targets, each of which may be relevant for cancer cachexia:

- Reduced catabolism, through non‐selective β receptor blockade.
- Reduced fatigue and thermogenesis, through central 5‐HT1a receptor antagonism.
- Increased anabolism, through partial β2 receptor agonism.^4^

This triad of both anticatabolic and pro‐anabolic pharmacological effects makes espindolol a unique and highly advantaged candidate for development in cancer cachexia. In addition to these effects on cancer cachexia, β blockers have very recently been shown to have a directly beneficial anticancer effect in certain forms of cancer.^5^

The potential use of espindolol for the treatment of cancer cachexia and age‐related muscle wasting has been well established in pre‐clinical models^6^, ^7^ that suggests that it may have beneficial effects upon multiple measures of the disease, including the potential to prolong survival. The favourable safety profile of espindolol at doses equivalent to those proposed in this clinical study has also been established in phase II clinical studies, one of which has been published.^8^ The ACT‐ONE trial was designed as a phase IIb study to test the hypothesis that high‐dose espindolol will be superior to placebo in reducing the rate of weight loss in advanced non‐curable CRC and NSCLC patients with cachexia and in secondary analyses to assess safety, tolerability, and effects on functional performance and quality of life instruments in these patients.

## Methods

### Trial design and oversight

The ACT‐ONE trial is a multicentre, randomized, double‐blind, parallel‐group, placebo‐controlled, dose‐finding phase II clinical study designed to evaluate the efficacy of two doses of espindolol administered over a 16‐week period in subjects with cachexia related to stage III and IV CRC and NSCLC. The study design has been published^9^, and the study was registered with ClinicalTrials.gov: NCT01238107.

Patients providing written informed consent were screened for eligibility and then received a 7‐day placebo [one tablet twice daily (bd)] run‐in. Eligible patients were then randomized on Day 0 in a 3:2:1 ratio to high‐dose espindolol (10 mg bd), placebo, or low‐dose espindolol (2.5 mg bd). All patients then attended for visits on Days 7, 14, 21, 28, 56, 84, 112, and 140. A procedure representative of the design of many studies of β blockers in chronic heart failure was followed to escalate the dose of study treatment within each patient over the first 2 to 4 weeks of dosing until either the target dose or the maximum tolerated dose for that patient was reached. The investigator was instructed to increase the dose as scheduled unless the patient demonstrated symptoms of intolerance after a given dose including resting heart rate dropping <50 beats per min, supine systolic blood pressure dropping <90 mmHg, the systolic blood pressure dropping >30 mmHg on assuming a standing position, or any significant new symptoms of postural hypotension or dizziness.

Standard of care chemotherapy, radiotherapy, nutrition, and supportive care was provided during the study as determined by their treating physician. Therapy was timed with the aim of minimizing the interference of chemotherapy on the study‐related functional assessments. All patients were followed for survival by visits or telephone contact up to the point of last patient last visit.

The study was designed, implemented, and overseen by a Steering Committee together with a representative of the sponsor, PsiOxus therapeutics (further detail concerning the trial management is available in the supplementary material). The protocol was approved by the institutional review board at each participating centre and conducted in accordance with the principles of the Declaration of Helsinki (1996), International Conference on Harmonisation Good Clinical Practice, local and national regulations, and as set forth in 21 EU Directive 2001/20/EC and GCP Directive 2005/28/EC. Written informed consent was provided by all patients prior to any study‐related procedures. The study design has been published, and the study was registered with ClinicalTrials.gov: NCT01238107.

### Study participants

Patients were recruited from 16 sites in three countries (India, Malaysia, and Germany). Eligible patients were aged between 25 to 80 years of age, with a life expectancy of greater than 3 months as judged by the treating physician and a confirmed diagnosis of either stage III or stage IV CRC not suitable for surgery or stage III or stage IV NSCLC not suitable for surgery. Patients were receiving (or had already received) a course of chemotherapy, with or without radiotherapy or surgery, with either a platinum‐based regimen for NSCLC or a 5‐fluorouracil or irinotecan‐based regimen for CRC. Patients suffered from cachexia because of the underlying cancer in the opinion of the investigator, with one of the following: a ≥5% documented weight loss in the previous 12 months, a subjective report of weight loss in the previous 12 months and a recorded body mass index (BMI) less than 20 kg/m^2^, or ongoing documented weight loss of at least 1 kg in the week prior to Day 0, or 1.25 kg in the 2 weeks prior to Day 0, or 1.5 kg in the 3 to 6 weeks prior to Day 0 provided that BMI was not more than 25 kg/m^2^. All patients reported at least two of the following: a subjective report of decreased muscle strength, a subjective report of fatigue, a subjective report of anorexia, or abnormal biochemistry [one or more of raised C‐reactive protein (CRP), low haemoglobin in the range <12 g/dL, or a low serum albumin in the range <3.2 g/dL]. Additional inclusion criteria included an Eastern Cooperative Oncology Group score of 0, 1, or 2, an ability to complete the study efficacy performance tests at the screening visit and with 6‐min corridor walk test (6MWT) distances at screening and the end of run‐in that differed by no more than 30% from each other. Patients also had to be at least 80% compliant during the run‐in period.

Key exclusion criteria included congestive heart failure, uncontrolled hypertension (with blood pressure >160/95 mmHg), pulse rate less than 68 beats per minute or high degree conduction defect on the electrocardiogram, a resting supine systolic blood pressure less than 100 mmHg, a history of bronchospasm and bronchial asthma, or a diagnosis of brain metastases. Other exclusions are listed in the supplementary material.

### Randomization and masking

Randomization was centralized in blocks, in a ratio 3:2:1 (high‐dose espindolol:placebo:low dose espindolol). Further detail is provided in the supplementary material.

### Outcome measures

Body weight was assessed at Days 0, 7, 14, 21, 28, 56, 84, 112, and 140 using a standard digital scale. The primary outcome measure was the difference in the rate (slope) of weight change between high‐dose espindolol and placebo between Days 0 and 112 expressed in kilogram per 4 weeks. The comparison between high‐dose, low‐dose, and placebo groups on the rate (slope) of weight change was assessed as a secondary measure.

The effect of treatment on safety (including adverse events) and other performance parameters were also assessed as secondary outcome measures. The following performance parameters were assessed in a fixed sequence, separated by rest periods and using standard procedures and equipment at Days 0, 28, 56, 84, and 112: hand grip strength (HGS)^10^ (modified to use a standard digital hand dynamometer), stair climbing power (SCP)^11^ (modified to account for the different stairway configurations at the investigational sites with the results analysed as a normalized power per kilogram), short physical performance battery (SPPB)^12^ test, and the 6MWT.^13^ Body composition was assessed by whole body dual‐energy X‐ray absorbitometry (DEXA) scanning at Days −1, 56, and 112. Quality of life was assessed using the EQ‐5D instrument (the official EQ‐5D translation for the subject's first language was used) at Days 0, 28, 56, 84, and 112. Overall survival data were collected until the end of the study.

### Statistical methods

The size of the primary analysis population for this study was planned conservatively at 132 patients randomized in a 3:2:1 ratio (high‐dose espindolol:placebo:low‐dose espindolol), based on an expected mean weight change per 4‐week period of −0.8 kg in the placebo group and a mean weight change per 4‐week period of 0 kg in the high‐dose espindolol group, a standard deviation of 1.2 kg per 4‐week period, an allocation ratio of 3:2 (high‐dose espindolol:placebo), and a two‐sided significance test for the rejection of the null hypothesis with a significance level *α* of 0.05 and a power of 85% (further detail of statistical methods are provided in the supplementary material). Patient recruitment was stopped after 87 patients were enrolled for organizational reasons, related to the lack of funds to continue. With this reduction in sample size, the power of the statistical tests is calculated to be 78% for the primary outcome and 63% for the secondary outcomes.

Comparisons between continuous efficacy variables and treatment were performed using an analysis of variance (ANOVA) model if the variable was normally distributed and using a non‐parametric Kruskall–Wallis test if it was not. Pairwise comparisons were performed using a Student's *t*‐tests or Wilcoxon tests. The relationship between categorical variables and treatment was analysed using a chi‐squared test or Fisher exact test, if applicable. The comparison between treatments was analysed using a linear mixed‐effect model for repeated measures with baseline value, treatment (i.e. the two active treatments and placebo), time (in units of 4 weeks), and interaction between treatment and time as fixed effects and subjects as a random effect. All pairwise differences of levels of the treatment effect were compared using the Bonferroni adjustment. Time‐to‐event data were analysed by a Kaplan‐Meier model by treatment group, and appropriate event rates using person‐time ‘at risk’ denominators were given. Bonferroni's adjustment was applied for multiple comparisons of the survival distribution functions corresponding to the treatment groups. A proportional hazards Cox regression model was applied to obtain hazard ratios and corresponding 95% confidence intervals (CIs). As per the statistical analysis plan, the primary and secondary efficacy outcomes were performed on the modified intent to treat (mITT) population (a subset of the ITT population who were at least 80% compliant through Day 28 per protocol). Supportive analyses were performed on the primary and key secondary outcomes for the ITT and according to protocol populations.

## Results

### Follow‐up and disposition of patients

**Figure 1:**
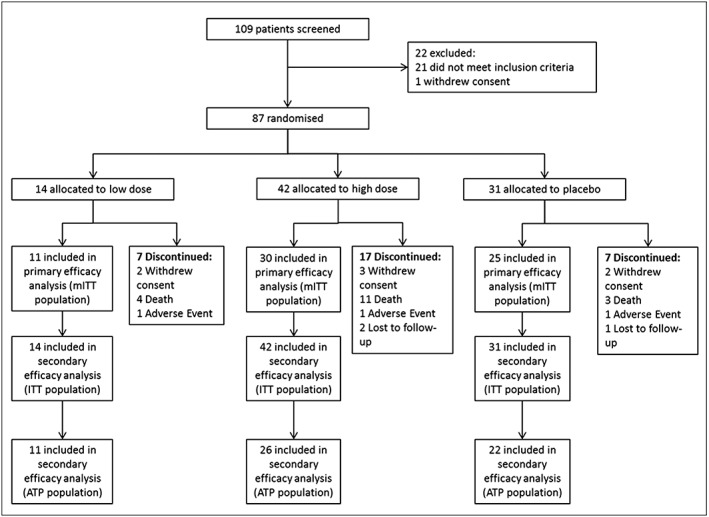
Study disposition and analysis populations. As reflects the severity of the cancers in this study, 18 deaths occurred and three discontinued because of adverse events. ITT, intention to treat population; mITT, modified intention to treat population; ATP, according to protocol population.

The number of patients screened, randomized, and analysed is shown in *Figure* 1. The baseline demographics of the randomized patients are summarized in Table 1. More detail on baseline measures, co‐morbidities, and prior and ongoing chemotherapy are documented in *Tables S4*, *S5*, and *S6* in the supplementary material. A total of 58 subjects presented with NSCLC and 29 with CRC; 69 subjects had metastatic disease at baseline: 44 with NSCLC and 25 with CRC. Most NSCLC subjects had received a platinum doublet (carboplatin or cisplatin) and most CRC subjects an oxaliplatin‐based chemotherapeutic regime prior to study entry. Compliance with study medication was high for all three treatment groups, with median percentage of tablets taken being 94.5, 95.5, and 97.6% in the low‐dose espindolol, high‐dose espindolol, and placebo group, respectively.

**Table 1:** Demographic and baseline clinical characteristics of the patients

	Placebo *N* = 31	Low dose 2.5 mg bd *N* = 14	High dose 10 mg bd *N* = 42
**Age** (mean ± SD, years)	55.0 ± 11.0	56.1 ± 12.2	59.3 ± 10.2
Gender (% female/% male)	40%/60%	29%/71%	31%/69%
Disease (% CRC/% NSCLC)	32%/68%	50%/50%	29%/71%
Stage (% IIIA/% IIIB/% IV)	7%/29%/65%	7%/7%/86%	7%/5%/88%
ECOG (% 0/% 1/% 2)	45%/39%/16%	29%/71%/0%	36%/55%/10%
Time from diagnosis (mean ± SD, years)	1.0 ± 1.3	1.6 ± 1.5	1.0 ± 1.0
BMI (mean ± SD, kg/m^2^)	20.0 ± 2.8	21.5 ± 4.2	20.1 ± 3.8
BMI <18.5 [number (%)]	5 (16%)	3 (21%)	19 (45%)
Anaemia [number (%)]	1 (3%)	0 (0%)	8 (19%)

BMI, body mass index (the weight in kilograms divided by the square of the height in metres); ECOG, Eastern Cooperative Oncology Group performance scale^14^.

Percentages are based on the number of patients randomized.

### Primary efficacy results

The multivariate analysis for the primary efficacy outcome of slope of absolute weight change over 16 weeks showed a highly statistically significant and clinically important effect for both the ITT and mITT populations (Table 2, top) with a weight gain of 0.54 kg/4 weeks for high‐dose espindolol compared with a weight loss of 0.21 kg/4 weeks for placebo in the mITT population. Similar findings were seen when the slopes of percentage weight change were calculated (Table 2, bottom).

**Table 2:** Multivariate analysis of the slope of weight change (ITT and mITT populations) for absolute weight changes (top) and percentage weight changes (bottom)

	ITT			mITT		
	Estimate	CI	*P* value	Estimate	CI	*P* value
Slopes of absolute weight changes (kg/4 weeks)						
High‐dose group	0.42	0.20, 0.64	<0.0001	0.54	0.38, 0.70	<0.0001
Placebo group	−0.37	−0.62, −0.11		−0.21	−0.37, 0.05	
LS means difference (high dose‐placebo)	1.14	0.71, 1.57	<0.0001	1.28	0.54, 2.03	0.0008
Slopes of percentage weight changes (%/4 weeks)						
High‐dose group	0.85	0.43, 1.26	<0.0001	1.04	0.75, 1.34	<0.0001
Placebo group	−0.66	−1.14, −0.18		−0.40	−0.70, −0.10	
LS means difference (high‐dose‐placebo)	2.24	1.43, 3.05	<0.0001	2.52	1.12, 3.92	0.0005

CI, confidence interval; ITT, intention to treat population; LS means, least squares means; mITT, modified intention to treat population.

### Secondary efficacy results

#### Weight change

**Figure 2:**
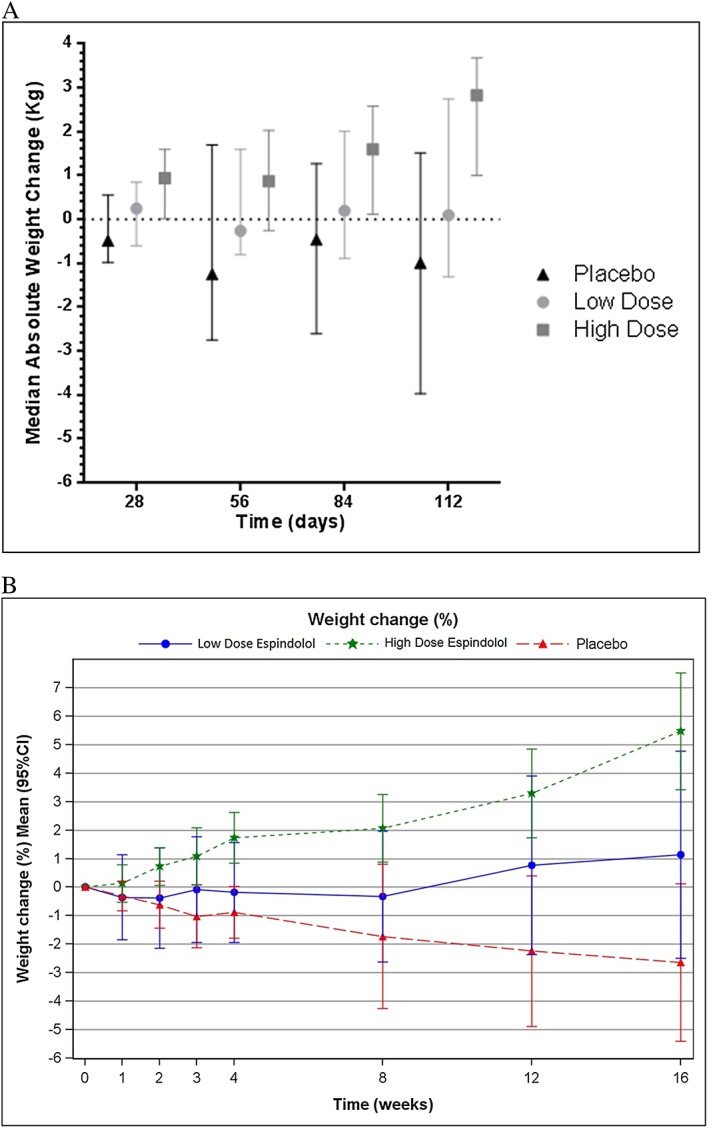
(*A*) Median absolute weight changes in the three randomized groups at Days 28, 56, 84, and 112 (mITT population). There appeared to be dose response in the treatment effect on body weight. There was a median weight gain in the high‐dose group at Day 112 of 2.83 kg (95% CI: 1.00, 3.68) compared with a weight loss of 0.99 kg (95% CI: −3.97, 1.52) in the placebo group and a weight gain of 0.10 kg (95% CI: −1.31, 2.75) in the low‐dose group. Similar trends were seen developing at earlier time points in the trial. (*B*) Relative change of weight (%) by visit (ITT population). Mean relative weight change shown as a percentage from baseline. CI, confidence interval.

The weight change results whether measured as median weight changes (*Figure* 2A), percentage weight changes (not shown), or the mean relative weight change as a percent from baseline (*Figure* 2B) all demonstrated a progressive dose‐related protection from ongoing weight loss with espindolol. The multivariate analysis for the slope of absolute and percentage weight change also shows a statistically significant difference in favour of the low dose vs. placebo group in the mITT population (not significant in the ITT population). The least squares means difference was 0.79 (95% CI: 0.28, 1.30; *P* < 0.01) for the absolute weight change and 1.43 (95% CI: 0.48, 2.38; *P* = 0.01) for the percentage weight change. There was no statistical difference in either population between the high‐dose and low‐dose groups for the slope of weight change.

#### Change of body composition according to dual‐energy X‐ray absorbitometry

**Figure 3:**
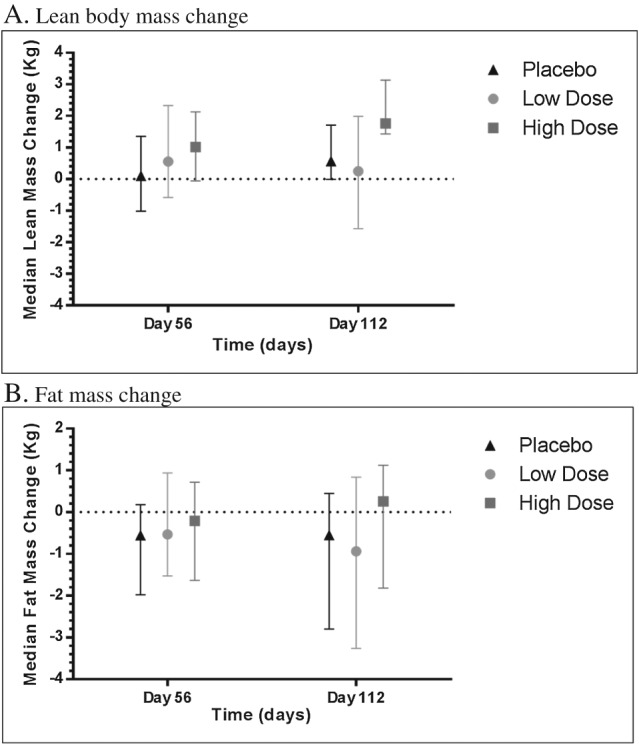
Median body composition change based on DEXA scan analysis (mITT population). (*A*) Lean body mass change. (*B*) Fat mass change. Body composition results for (*A*) lean body mass and (*B*) fat mass as derived from the DEXA scans at Days 56 and 112. Data shown is median change from baseline with 95% CI.

In the mITT population, there was an absolute median lean body mass (LBM) gain in the high‐dose espindolol group at Day 112 of 1.76 kg (95% CI: 1.43, 3.18) compared with a gain of 0.57 kg (95% CI: −0.01, 1.71) in the placebo group and a gain of 0.25 kg (95% CI: −1.57, 1.99) in the low‐dose espindolol group (*Figure* 3A). The changes between high‐dose and placebo changes were statistically significant (*P* = 0.012) and likewise in the ITT population (*P* = 0.036). The difference in the LBM change was not statistically significant either between the high‐dose and low‐dose groups or between the low‐dose and placebo groups.

There was an absolute median fat mass gain in the high‐dose espindolol group at Day 112 of 0.26 kg (95% CI: −1.82, 1.12) compared with a loss of 0.55 kg (95% CI: −2.80, 0.45) in the placebo group and a loss of 0.94 kg (95% CI: −3.26, 0.84) in the low‐dose espindolol group (*Figure* 3B). These changes showed a numerical trend towards benefit but were not statistically significant between high‐dose and placebo groups in either the mITT population (*P* = 0.56) or ITT population (*P* = 0.071) but certainly suggest a preservation of fat mass despite ongoing cachexia.

#### Performance tests

**Figure 4:**
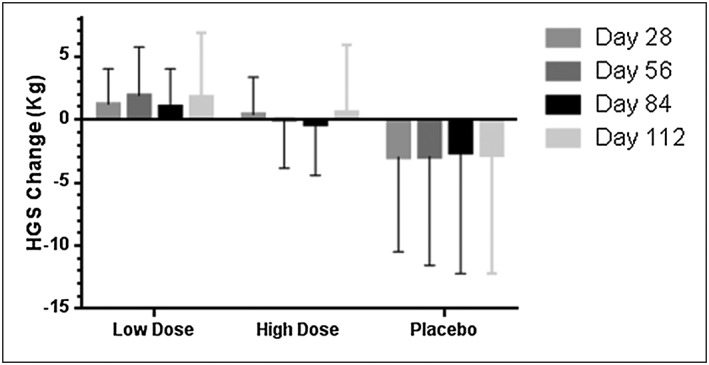
Effects on hand grip strength (mITT population). High‐dose espindolol was significantly superior to placebo, *P* = 0.0134.

There was consistent trend towards benefit for the high‐dose espindolol group when compared with placebo in the four performance tests analysed, most notably for HGS (*Figure* 4). In the multivariate analysis, the slope of absolute change in HGS showed a statistically significant benefit of both high‐dose and low‐dose espindolol compared with placebo in the mITT population (Table 3) and also in the ITT population. Whilst the results for the slope of percentage change in 6MWT, SCP, and SPPB were all directionally in favour of high‐dose espindolol vs. placebo, these comparisons were not statistically significant (results not shown, available in supplementary material).

**Table 3:** Multivariate analysis of hand grip strength performance tests (mITT population)

	Absolute change			Percentage change		
	LS means differences			LS means differences		
	Estimate	CI	*P* value	Estimate	CI	*P* value
HGS						
Low dose‐high dose	1.80 (1.08)	(−0.33, 3.94)	0.2936	−6.38 (4.88)	(−15.99, 3.23)	0.5762
Low dose‐placebo	4.16 (1.10)	(2.00, 6.33)	0.0006	−0.03 (4.96)	(−9.81, 9.75)	1.0000
High dose‐placebo	2.36 (0.82)	(0.74, 3.98)	0.0134	6.35 (3.69)	(−0.92, 13.62)	0.2592

Baseline value of HGS is the value at Day 0. Changes in HGS value were computed with respect to its baseline value.

HGS, hand grip strength.

#### Quality of life measures

There were no trends or significant differences in the visual analogue score or the EQ‐5D Index. The results are presented in *Figure* S1 in the supplementary material.

#### Overall survival

**Figure 5:**
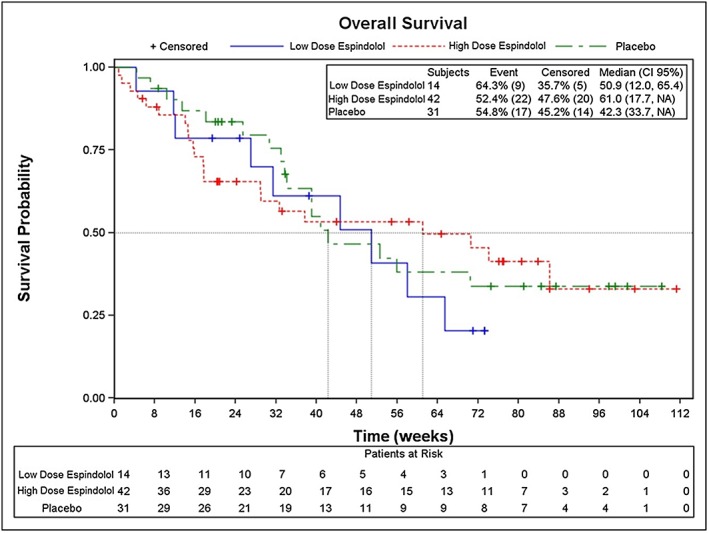
Overall survival. Kaplan–Meier curves (ITT population).

The median overall survival was longer for the high‐dose espindolol group (61.0 weeks) compared with both the low‐dose espindolol group (50.9 weeks) and the placebo group (42.3 weeks) (*Figure* 5). These survival differences were not statistically significant.

### Safety results

All commonly reported treatment emergent events (occurring in more than 10% subjects in a treatment group) are listed in a *Table* S1 in the supplementary material. The most frequently reported treatment emergent events overall were anaemia (16.1%), cough (12.%), and dyspnoea (10.3%). Dyspnoea was more prevalent for the high‐dose espindolol group (19.1%) compared with the placebo (3.2%) and low‐dose espindolol (0%) groups. Anaemia was more prevalent in both the high‐dose espindolol (19.1%) and low‐dose espindolol (28.6%) groups compared with the placebo group (6.5%). All but one reported cases of dyspnoea occurred in the NSCLC cohort. Dyspnoea might be expected for some patients with a background of lung cancer receiving a β blocker because of the increased likelihood of chronic lung disease in this group. The imbalance of anaemia may be related to the baseline status of the patients (nine patients in total were noted to have anaemia at baseline of which eight were subsequently randomized to high‐dose espindolol and one to placebo). Serious treatment emergent adverse events are summarized in *Table* S2 in the supplementary material. There appeared to be no pattern to these events.

## Discussion

Attempts to treat cachexia have to date focused largely on anabolic or nutritional supplementation therapies. The most convincing published study prior to this report of the ACT‐ONE trial was a phase II study of enobosarm (GTx‐024; GTx, Memphis, TN, USA), a selective androgen receptor modulator performed in patients with the closely related clinical condition of cancer‐induced muscle wasting.^15^ In patients with NSCLC (stages II, III, or IV), CRC (stages II, III, or IV), non‐Hodgkin lymphoma, chronic lymphocytic leukaemia, or breast cancer (stages III, or IV) with at least 2% weight loss, there was a statistically significant increase in total LBM from baseline, assessed by DEXA scanning, in both enobosarm groups (enobosarm 1 mg: median 1.5 kg increase, range −2.1 to 12.6; *P* = 0.0012 and enodosarm 3 mg: median 1.0 kg increase, −4.8 to 11.5; *P* = 0.046) but not in the placebo group (median 0.02 kg, range −5.8 to 6.7; *P* = 0.88). Two phase III trials of enobosarm in cancer‐related cachexia showed inconsistent results, however. The co‐primary endpoints in both studies were a responder analysis in LBM and SCP. In one study (514 study), LBM was improved, whereas SCP was not. In the second trial (505), neither was improved. These trials have only been presented at conferences and not yet in a peer‐reviewed publication.^16^

Anamorelin, an oral ghrelin mimetic, was tested in several trials. In a cross‐over study in 16 patients with cancer‐related cachexia, anamorelin 50 mg/day over 3 days significantly increased body weight compared with placebo (0.77 kg vs. −0.33 kg), and appetite was reported as being increased.^17^ In another set of studies investigating anamorelin for patients with cancer cachexia^18^ (74 patients analysed, 44 in the anamorelin group), over 12 weeks LBM decreased by 0.2 kg in patients on placebo, whereas it increased by 1.9 kg in patients on anamorelin [treatment effect 2.09 kg (95% CI: 0.94–3.25]; *P* = 0.0006). In this trial programme, anamorelin caused a 0.5 kg weight gain, whereas patients on placebo lost about 1.8 kg. Changes in body weight were strongly related to changes in lean mass (*r* = 0.72, *P* = 0.0001). The treatment was also associated with increased non‐dominant HGS (treatment effect 2.59 kg; *P* < 0 · 02) associated with increased non‐dominant HGS (treatment effect 2.59 kg; *P* < 0.02). In the paired phase III trials (Romana 1 and 2), anamorelin improved only one of the two co‐primary endpoints (LBM but not HGS) in patients with cancer‐related cachexia.^19^

L‐Carnitine supplementation has also shown activity in one study of 72 patients with advanced pancreatic cancer and weight loss. During treatment, BMI increased by 3.4 ± 1.4% with L‐Carnitine and decreased by −1.5 ± 1.4% in the placebo group (*P* < 0.05).^20^ One further small study^21^ has investigated the use of a nutritional supplementation regime, Ethanwell/Ethanzyme (EE) enriched with omega‐3 fatty acids, micronutrients, and probiotics, compared with control (Isocal) on BMI (rather than functional endpoints) in 68 patients with head and neck cancer‐related cachexia. The study was negative, but a subgroup analysis of those with entry BMI <19 mg/m^2^ showed that the EE regimen significantly increased body weight and maintained higher serum albumin and pre‐albumin levels compared with control (*P* < 0.05) over 3‐month treatment. No significant effect was seen in a small study of melatonin closed for reasons of futility after only 48 patients had been recruited.^22^

There have been fewer trials employing an anticatabolic approach. Three small studies of TNF‐α inhibitors etanercept,^23^ infliximab,^24^ and thalidomide^25^ failed to show benefits. In an earlier and now abandoned clinical trial programme, the ACE inhibitor imidapril had been studied in 200 patients with one of three cancer types, and improvement in body weight was reported in two (CRC and NSCLC) but not in the third type studied (pancreatic cancer) nor in the pre‐specified analysis of all three cancer types taken together.^26^

### Summary of the clinical endpoints of ACT‐ONE

The ACT‐ONE trial is one of the very few positive phase II studies to date in the field of cachexia research. The primary endpoint showed a highly statistically significant (*P* < 0.0001) and clinically relevant effect with a positive weight slope of 0.54 kg/4 weeks (95% CI: 0.38–0.70) for the high‐dose espindolol group compared with the placebo group with a negative weight slope of −0.21 kg/4 weeks (95% CI: −0.37, −0.05). The difference in weight change between the two groups of 0.75 kg every 4 weeks equates to a difference of 3 kg over the 16‐week duration of the study. This is the largest effect seen in any placebo‐controlled clinical trial in cancer‐related cachexia. The pharmacological profile of espindolol may explain a particular benefit seen in this study: statistically significant body composition changes with increases in fat‐free mass and no loss of fat mass suggesting that unlike other agents, the muscle mass is not increased at the expense of body energy stores in the form of adipose tissue. The fact that all functional secondary endpoints HGS, SCP, and 6MWT were all directionally in favour of high‐dose espindolol vs. placebo was another feature suggestive of a potentially important role for espindolol in the treatment of cancer‐related cachexia. This combination of a large increase in body weight, derived from a gain in muscle with no loss of fat, and a significant increase in HGS, a meaningful test of functional capacity, all suggest a positive role for espindolol that warrants further assessment in a subsequent phase III clinical trial. The relative preservation of fat mass may be related to known beta‐blocker effects such as fat mass increase seen in CHF patients (know to be at high risk of cachexia) when commencing a beta‐blocker.^27^ It is interesting to speculate why espindolol in this trial significantly increased HGS whereas the larger Romana 1 and 2 trials failed (refer to Garcia et al., 2013^17^). This could be related to the details of the methodology. We performed three HGS test with each arm and then a four test on the stronger arm, taking the highest value as the study measurement, whereas the Romana trial programme used only a single measurement in the non‐dominant arm, which may have added considerable extra variability to their measurement of HGS.

### Limitations

This was a relatively small phase II trial with a fairly heterogeneous population, so the data will need to be confirmed in a phase III trial. This was exacerbated by early cessation of patient recruitment for operational reasons. The small numbers in the low‐dose espindolol group preclude any investigation of dose response. There was an imbalance at baseline in anaemia (also seen for some other markers of severity) that indicates by chance that the high‐dose espindolol group may have recruited ‘sicker’ patients. Patients with BMI <18.5 kg/m^2^ at baseline, the most wasted, showed an imbalance between groups; five were randomized to placebo (16.1% of placebo patients), three to low‐dose espindolol (21.4% of low‐dose patients), and 19 to high‐dose espindolol (45.2% of high‐dose patients), so it is even more remarkable that the beneficial effects in protection from weight loss and improvement in HGS was more marked in these patients.

## Conclusions

The ACT‐ONE study has demonstrated the efficacy and dose response of espindolol at a dose of 10 mg bd in patients with cachexia because of two common forms of cancer: NSCLC and CRC. Over 16 weeks, efficacy has been demonstrating in both reducing weight loss and promoting weight gain, associated with significant improvements in HGS, with no adverse safety signals. Beneficial effects were seen on lean mass with a trend for fat tissue. The difference of slope of weight changes was highly statistically significant between high‐dose espindolol and placebo groups consistently across all analysis populations

Data from this study will allow the design and conduct of appropriate phase III clinical studies to confirm the utility of espindolol for treatment of cachexia related to these two common cancer types.

## Funding

Sponsored by PsiOxus therapeutics

## Conflict of interest

Drs Coats and Anker report that they are scientific founders of PsiOxus, the sponsor, and have received equity (less than 1% of diluted share capital) and receiving consultancy fees, royalties, and research grants. Dr Beadle, Dr Brown, and Julia Tilson report being employees of PsiOxus having received equity and salary. Dr von Haehling reports receiving patient fees from PsiOxus during the conduct of the study, Drs Ho and Prabhash have nothing to disclose.

## Supporting information


**Table S1.** Multivariate analysis of performance tests (ITT population)
**Table S2.** Treatment emergent adverse events
**Table S3.** Treatment emergent serious adverse events
**Table S4.** Baseline outcome measures in the ITT population. Figures shown are median values
**Table S5.** Major comorbidities in the ITT population. Figures shown are actual values, with % in parentheses
**Table S6.** Prior and ongoing chemotherapies between treatment arms in the ITT population. Figures shown are actual values, with % in parentheses
**Figure S1.** EQ‐5D index—ITT population

Supporting info item
