# Supplementary material for: Espindolol for the treatment and prevention of cachexia in patients with stage III/IV non‐small cell lung cancer or colorectal cancer: a randomized, double‐blind, placebo‐controlled, international multicentre phase II study (the ACT‐ONE trial)
Source: J Cachexia Sarcopenia Muscle. 2016 Jul 1;7(3):355–65. doi: 10.1002/jcsm.12126 (PMC4929828; doi:10.1002/jcsm.12126)
Supplement: Supplementary file 1 — Supporting info item [file JCSM-7-355-s001.docx]

Supplementary material:

### Trial Oversight

The study was designed, implemented and overseen by a Steering Committee together with a representative of the sponsor, PsiOxus therapeutics. Two independent Medical Monitors reviewed all safety surveillance data, throughout the study and made recommendations regarding further conduct of the study to the Steering Committee. On-site monitoring of sites was conducted by Veeda Oncology and MakroCare (India), Klinsel (Malaysia) and Pivotal (Germany). Data collection and data management was conducted by Veeda Oncology and Pivotal. Statistical analysis was conducted by Pivotal.

The protocol was approved by the institutional review board at each participating centre and conducted in accordance with the principles of the Declaration of Helsinki (1996), International Conference on Harmonisation Good Clinical Practice, local and national regulations and as set forth in 21 EU Directive 2001/20/EC and GCP Directive 2005/28/EC. Written informed consent was provided by all patients prior to any study related procedures. The study design has been published^[[1]](#endnote-1)^ and the study was registered with ClinicalTrials.gov: NCT01238107. The manuscript was prepared and submitted for publication by the Steering Committee which had access to the study data and vouches for the accuracy and completeness of the reported analyses.

**Exclusion Criteria**

Key exclusion criteria included congestive heart failure, uncontrolled hypertension (with blood pressure >160/95 mm Hg), use of a pacemaker, implantable defibrillator, or internalised metal stent, pulse rate less than 68 beats per minute or high degree conduction defect on the electrocardiogram, a resting supine systolic blood pressure less than 100 mm Hg, a history of bronchospasm and bronchial asthma or a diagnosis of brain metastases. Other exclusions included pregnancy or lactation, a ≥20% weight loss in the previous 3‑months or a BMI of less than 16 kg/m^2^, mechanical obstruction of the alimentary canal, intractable vomiting, any history or clinical evidence of any hyperthyroidism, cirrhosis, hepatic failure, human immunodeficiency virus (HIV), renal failure (a serum creatinine > 250 μmol/l or > 2·83 mg/dl at screening), active tuberculosis, or any physical, medical, socioeconomic or other non-cancer related cause for simple starvation, muscle wasting or weight loss. Other exclusions were current or planned treatment with any oral adrenal corticosteroids, βadrenergic blockers, on-dihydropyridine calcium antagonists (e.g. Verapamil, diltiazem), α‑adrenergic blockers, ivabradine, 5‑HT agonists or antagonists e.g. serotonin reuptake inhibitors (SSRI’s), (short‑term use around the time of chemotherapy were acceptable), monoamine oxidase inhibitors (MAOI’s), β agonists, (short term or on-and-off use of inhaled bronchodilators were acceptable), amiodarone, megestrol, anabolic steroids or any other prescription medication intended to increase appetite or to treat unintentional weight loss, or treatment with any investigational drug therapy within 28 days prior to the screening visit.

### Randomisation and Masking

Randomisation was centralized via a list prepared using the SAS program, Version 9.2 in blocks, in a ratio 3:2:1 (high dose espindolol: placebo: low dose espindolol:). The placebo formulation was identical in appearance to the espindolol formulations. The sponsor, study personnel, sites, and patients were all blinded to treatment assignments. At screening, if the subject met all eligibility criteria, site personnel completed the Subject Randomisation Form and sent it to the CRO Project Manager who assigned the randomisation number for that subject. A sealed copy of the randomisation code was provided to the nominated person responsible for packaging and one sealed copy was kept at the CRO biometrics department.

### Statistical Methods

The size of the primary analysis population for this study was planned conservatively at 132 patients randomised in a 3:2:1 ratio (high dose espindolol: placebo: low dose espindolol), based on an expected mean weight change per 4-week period of -0·8 kg in the placebo group and a mean weight change per 4-week period of 0 kg in the high dose espindolol group; a standard deviation of 1·2 kg per 4-week period; an allocation ratio of 3:2 (high dose espindolol : placebo) and a two-sided significance test for the rejection of the null hypothesis with a significance level α of 0.05; a power of 85%. Background assumptions were based the results of the Randomised Clinical Trial of Adenosine 5-Triphosphate in Patients With Advanced Non-Small-Cell Lung Cancer^[[2]](#endnote-2)^, and two similar trials^[[3]](#endnote-3),^^[[4]](#endnote-4)^. Allowing for 20% of recruited patients not complying with the criteria for inclusion, 66 patients were planned for the high dose espindolol group, 22 patients for the low dose espindolol group, and 44 patients for the placebo group. Patient recruitment was stopped after 87 patients were enrolled for organisational reasons. With this reduction in sample size, the power of the statistical tests is calculated to be 78% for the primary outcome and 63% for the secondary outcomes.

Statistical analyses were performed according to the statistical analysis plan that was approved by the Steering Committee prior to study unblinding. No interim analyses were performed. The final analyses were performed on data entered into Oracle Clinical and exported to SAS datasets, and all analyses and reporting were performed using SAS® for Windows Version 9.2 or higher.

All outcome measures were summarized descriptively. Comparisons between continuous efficacy variables and treatment were performed using an ANOVA model if the variable was normally distributed, and using a non-parametric Kruskall-Wallis test if it was not. Pairwise comparisons were performed using a Student t‑tests or Wilcoxon-tests. The relationship between categorical variables and treatment was analysed using a Chi-square test or Fisher exact test, if applicable. The comparison between treatments was analysed using a linear mixed-effect model for repeated measures with baseline value, treatment (i.e. the two active treatments and placebo), time (as unit of 4 weeks), interaction between treatment and time as fixed effects and subjects as random effect. All pairwise differences of levels of the Treatment effect were compared using the Bonferroni adjustment. Time-to-event data were analysed by a Kaplan-Meier model by treatment group and appropriate event-rates using person-time “at risk” denominators were given. Bonferroni’s adjustment was applied for multiple comparisons of the survival distribution functions corresponding to the treatment groups. A proportional-hazards Cox regression model was applied to obtain Hazard Ratios and corresponding 95% CIs.

The primary and secondary efficacy outcomes were performed on an ITT population (subjects who were randomised into the study and received at least one dose of study treatment). A supportive analyses on a modified ITT population (a subset of the ITT population who were at least 80% compliant through Day 28 per protocol) was performed on the primary and key secondary outcomes.

All secondary variables were assessed according to a pairwise comparison: High-dose vs Placebo; Low-dose vs Placebo; and High-dose vs Low-dose. The p values have all been adjusted for multiple comparisons using the Bonferroni correction. Secondary analyses were performed on weight change in the three groups and the effect of the two doses of espindolol (10 mg bd and 2.5 mg bd) in comparison with placebo over a sixteen week period on other end-points of interest, including function, quality of life and body composition end-points.

The summary statistics of: tissue (% fat), region (% fat), total mass (kg), tissue mass (g), fat mass (g), lean mass (g), BMC (g) obtained and their change from baseline, were all analysed for each treatment group on each visit. The change on each visit expressed as the absolute change from baseline, and as the % change from baseline (calculated as the difference and as the percent variation) was analyzed using a linear mixed-effect model for repeated measures.

From the EQ-5D questionnaire summary statistics of the each sub-scale (mobility, self-care, usual activities and Pain/Discomfort, Anxiety/Depression), the total score and Visual Analog Scale (VAS), their absolute change from baseline, and the differences between treatments, were analysed, for each treatment group on each visit. The comparison between treatments in QOL scores over study period were analyzed using a linear mixed-effect model for repeated measures. Changes in VAS value were computed with respect to its baseline. The multivariate analysis for the slope of absolute change for EQ-5D did not show any significant differences between the treatment groups. The EQ-5D Index was calculated by using the UK TTO EQ-5D-3L value set. This value set has been derived using TTO valuation techniques and reflects the opinions of the UK general population. No value sets were available for the populations actually studied. The EQ-5D index for the ITT population showed no statistically significant changes.

Missing data were imputed as follows:

- For patients who died or discontinued treatment due to an adverse event or death before 4 weeks, missing data were imputed using the worst observed slope of weight change

- For the remaining patients, missing data were imputed using the Mixed-Effect Mode for Repeated Measure (MMRM) method. ^[[5]](#endnote-5)^.

After the imputation process, the analyses were performed using the MMRM method. MMRM analysis information from the observed data is used via the within-patient correlation structure to provide information about the unobserved data, but the missing data are not explicitly imputed.

Results:

Full performance test results table (enlarged version of table 3)

Table A Multivariate Analysis of Performance Tests (ITT Population)

|  | Absolute Change | | | Percentage Change | | |
| --- | --- | --- | --- | --- | --- | --- |
|  | LS Means Differences | | | LS Means Differences | | |
|  | Estimate | CI | p-value | Estimate | CI | p-value |
| **HGS** |  |  |  |  |  |  |
| Low dose-high dose | 1.11 (1.00) | (-0.85, 3.08) | 0.7942 | 4.61 (6.18) | (-7.56, 16.78) | 1.0000 |
| Low dose-placebo | 3.86 (1.03) | (1.84, 5.89) | 0.0007 | 13.17 (6.42) | (0.52, 25.81) | 0.1240 |
| High dose-placebo | 2.75 (0.77) | (1.24, 4.26) | 0.0012 | 8.56 (4.83) | (-0.95, 18.07) | 0.2329 |
| **SCP** |  |  |  |  |  |  |
| Low dose-high dose | -3.53 (14.31) | (-32.07, 25.01) | 1.0000 | -5.40 (10.72) | (-26.51, 15.72) | 1.0000 |
| Low dose-placebo | 10.29 (14.69) | (-19.01, 39.59) | 1.0000 | 2.94 (11.01) | (-18.74, 24.62) | 1.0000 |
| High dose-placebo | 13.82 (11.15) | (-8.42, 36.06) | 0.6583 | 8.34 (8.36) | (-8.13, 24.80) | 0.9592 |
| **6MWT** |  |  |  |  |  |  |
| Low dose-high dose | 0.26 (25.39) | (-50.36, 50.89) | 1.0000 | -5.06 (7.63) | (-20.27, 10.15) | 1.0000 |
| Low dose-placebo | 20.40 (26.22) | (-31.86, 72.67) | 1.0000 | 4.56 (7.88) | (-11.15, 20.26) | 1.0000 |
| High dose-placebo | 20.14 (19.68) | (-19.09, 59.37) | 0.9285 | 9.62 (5.93) | (-2.20, 21.43) | 0.3274 |
| **SPPB** |  |  |  |  |  |  |
| Low dose-high dose | -0.28 (0.53) | (-1.33, 0.77) | 1.0000 | -3.27 (4.87) | (-12.99, 6.45) | 0.5048 |
| Low dose-placebo | 0.21 (0.55) | (-0.88, 1.30) | 1.0000 | 1.73 (5.05) | (-8.34, 11.80) | 0.7326 |
| High dose-placebo | 0.49 (0.41) | (-0.33, 1.31) | 0.7157 | 5.00 (3.79) | (-2.55, 12.55) | 0.1909 |

Legend to table A:

HHGS=hand grip strength, SCP=stair climbing power, 6MWT=six minute walk test, SPPB=short physical performance battery test

Baseline value of HGS is the value at day 0. Changes in HGS value were computed with respect to its baseline value.

Table B Treatment Emergent Adverse Events

| **Preferred Term** | **Number (Percentage) of Patients with Event** | | | |
| --- | --- | --- | --- | --- |
|  | **Espindolol** | | **Placebo (N=31)** | **Total**  **(N=87)** |
|  | **Low dose (N=14)** | **High dose (N=42)** |  |  |
| Anaemia | 4 (28.6%) | 8 (19.0%) | 2 (6.5%) | 14 (16.1%) |
| Cough | 2 (14.3%) | 6 (14.3%) | 3 (9.7%) | 11 (12.6%) |
| Dyspnoea | 0 | 8 (19.0%) | 1 (3.2%) | 9 (10.3%) |
| Vomiting | 4 (28.6%) | 2 (4.8%) | 2 (6.55) | 8 (9.2%) |
| Blood alkaline phosphatase increased | 2 (14.3%) | 4 (9.5%) | 1 (3.2%) | 7 (8.0%) |
| Nausea | 2 (14.3%) | 2 (4.8%) | 2 (6.5%) | 6 (6.9%) |
| Constipation | 0 | 1 (2.4%) | 4 (12.95) | 5 (5.7%) |
| Lethargy | 2 (14.3%) | 1 (2.4%) | 0 | 3 (3.4%) |
| Fatigue | 2 (14.3%) | 0 | 0 | 2 (2.3%) |

Legend to Table B - Treatment Emergent Adverse Events*

*All adverse events which occurred in >10% patients in any treatment group by preferred term are summarised. Adverse events were coded using MedDRA Version 15.1

Table C Treatment Emergent Serious Adverse Events

| **Preferred Term** | **Number (Percentage) of Patients with SAE** | | | |
| --- | --- | --- | --- | --- |
|  | **Espindolol** | | **Placebo (N=31)** | **Total**  **(N=87)** |
|  | **Low dose (N=14)** | **High dose (N=42)** |  |  |
| Dyspnoea | 0 | 4 (9.5%) | 0 | 4 (4.6%) |
| Sepsis | 0 | 1 (2.4%) | 2 (6.5%) | 3 (3.4%) |
| Acute myocardial infarction | 1 (7.1%) | 0 | 0 | 2 (2.3%) |
| Diarrhoea | 1 (7.1%) | 0 | 1 (3.2%) | 2 (2.3%) |
| Neoplasm progression | 1 (7.1%) | 1 (2.4%) | 0 | 2 (2.3%) |
| Abdominal pain | 0 | 0 | 1 (3.2%) | 1 (1.1%) |
| Anal fissure | 0 | 1 (2.4%) | 0 | 1 (1.1%) |
| Ascites | 0 | 1 (2.4%) | 0 | 1 (1.1%) |
| Bronchospasm | 0 | 1 (2.4%) | 0 | 1 (1.1%) |
| Femoral neck fracture | 0 | 1 (2.4%) | 0 | 1 (1.1%) |
| Dehydration | 1 (7.1%) | 0 | 0 | 1 (1.1%) |
| Lower respiratory tract infection | 0 | 0 | 1 (3.2%) | 1 (1.1%) |
| Melaena | 1 (7.1%) | 0 | 0 | 1 (1.1%) |
| Monoparesis | 1 (7.1%) | 0 | 0 | 1 (1.1%) |
| Neutropenic sepsis | 0 | 1 (2.4%) | 0 | 1 (1.1%) |
| Pleural effusion | 0 | 0 | 1 (3.2%) | 1 (1.1%) |
| Pneumonia | 0 | 0 | 1 (3.2%) | 1 (1.1%) |
| Sudden death | 0 | 1 (2.4%) | 0 | 1 (1.1%) |

Legend to Table C - Treatment Emergent Serious Adverse Events*.

*Adverse events were coded using MedDRA Version 15.1. SAE=serious adverse event

Table D: Baseline Outcome Measures in the ITT population. Figures shown are median values.

| Baseline Outcome Measures | Low Dose Espindolol | High Dose Espindolol | Placebo |
| --- | --- | --- | --- |
| Body Weight (kg) | 51.56 | 50.24 | 51.30 |
| 6MWT (m) | 412.25 | 378.25 | 379.25 |
| HGS (kg) | 24.13 | 22.98 | 27.20 |
| SCP (Power/kg) | 153.00 | 124.29 | 131.87 |
| SPPB (Total score) | 10.75 | 10.50 | 10.25 |

Legend to table D: ITT: Intention to Treat; 6MWT: 6 minute walk test; HGS: hand grip strength; SCP: stair climbing power; SPPB: short physical performance battery.

Table E: Major comorbidities in the ITT population. Figures shown are actual values, with % in parentheses.

| Co-morbidities | Low Dose Espindolol  (N=14) | High Dose Espindolol  (N=42) | Placebo  (N=31) |
| --- | --- | --- | --- |
| Heart Failure | 0 (0.00) | 1 (2.38) | 0 (0.00) |
| COPD | 1 (7.14) | 0 (0.00) | 0 (0.00) |
| Fatigue | 4 (28.57) | 10 (23.81) | 6 (19.35) |
| Hypertension | 4 (28.57) | 4 (9.52) | 1 (3.23) |
| Renal Impairment | 0 (0.00) | 0 (0.00) | 1 (3.23) |

Legend to table E: ITT: Intention to Treat; COPD: Chronic obstructive pulmonary disease.

Table F: Prior and ongoing chemotherapies between treatment arms in the ITT population. Figures shown are actual values, with % in parentheses.

|  | Low Dose Espindolol  (N=14) | High Dose Espindolol  (N=42) | Placebo  (N=31) |
| --- | --- | --- | --- |
| Patients with previous chemotherapy | 13 (92.86) | 41 (97.62) | 29 (93.55) |
| Patients with ongoing chemotherapy* | 8 (57.14) | 27 (64.29) | 18 (58.06) |

* Patients receiving anti-tumour therapy during the study, or had received in the 2-4 weeks prior to study entry.

Figure A EQ-5D Index –ITT Population


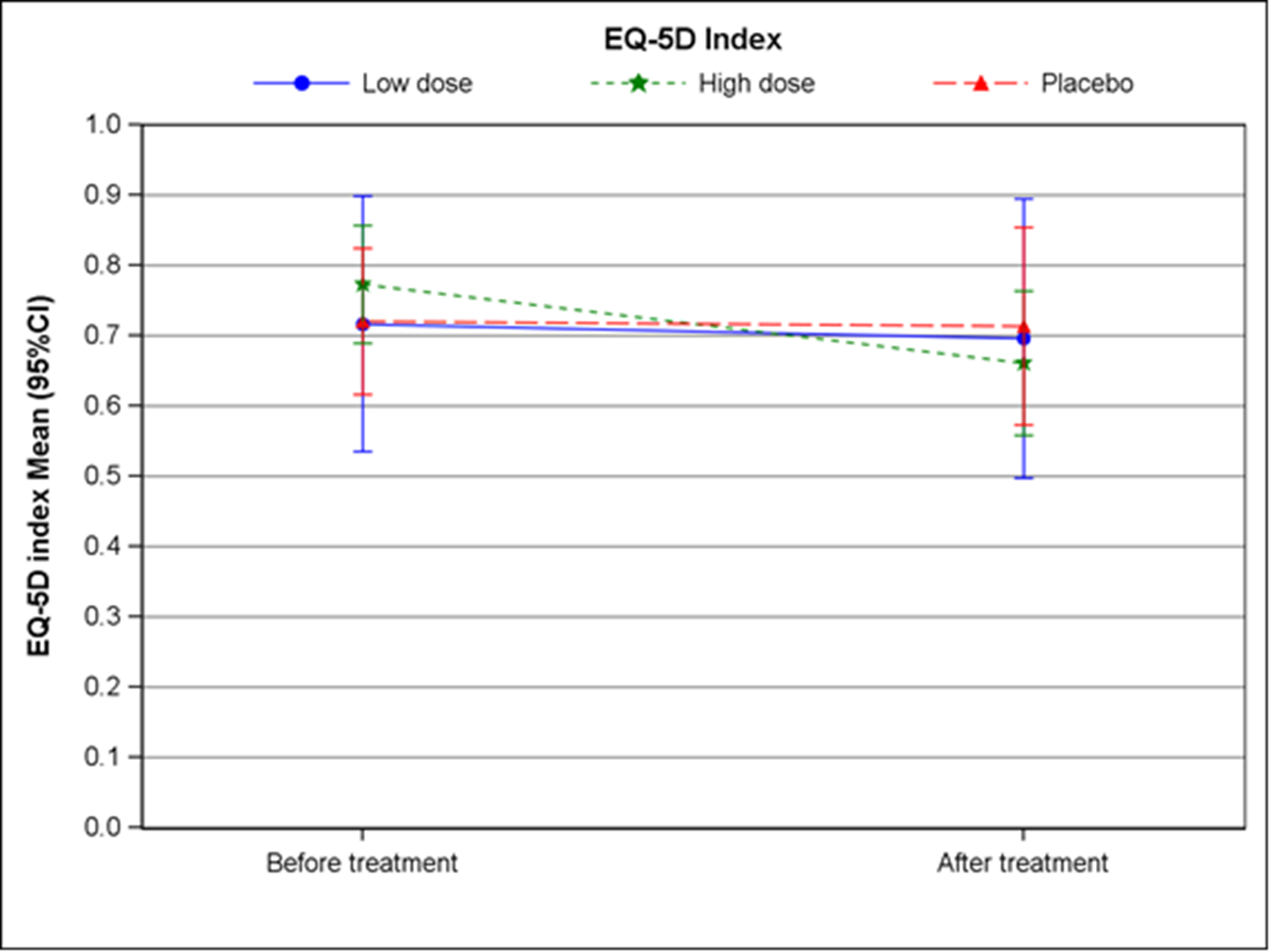


Legend to Figure A - The EQ-5D Index was calculated by using the UK TTO EQ-5D-3L value set. This value set has been derived using TTO valuation techniques and reflect the opinion of the UK general population. No value sets were available for the populations actually studied. Figure A shows EQ-5D index for the ITT population and which shows no statistically significant difference ebtween

**APPENDICES:**

The study was sponsored by PsiOxus Therapeutics

Acknowledgements:

We thank the investigators, study coordinators, nurses, and other clinical staff at all our investigative sites and especially all patients recruited into the ACT-ONE trial.

The members of the ACT-ONE study group are as follows:

Steering Committee: Andrew J Stewart Coats and Stefan D Anker (co-chairs), John Beadle,

Independent Medical Monitors: Aminah Jatoi and Raghunadharao Digumarti

Investigators/Institutions where the ACT-ONE study was performed:

India:

Dr. Kumar Prabhash - Tata Memorial Hospital

Dr. Niraj Bhatt - Kailash Cancer Hospital and Research Centre

Dr. Chiramana Haritha - M S Patel Cancer Centre, Shree Krishna Hospital

Dr. Minish Jain - Noble Hospital, Pune

Dr. Venkatesan Srinivasan - Dr Kamakshi Memorial Hospital

Dr. Shailesh Bondarde - Shatabdi Super Speciality Hospital

Dr. Rajnish Vasant Nagarkar - Curie Manavta Cancer Centre

Dr. Sandip Shah - Vedanta Institute of Medical Sciences

Malaysia:

Dr. Ho Gwo Fuang - Universiti Malaya Medical

Dr. Irfhan Ali Bin Hyder Ali - Penang General Hospital

Dr. Biswa Mohan Biswal - Hospital Universiti Sains Malaysia

Dr. Sangeetha Poovaneswaran - Tuanku Ja’afar Hospital

Dr. Mohd Zailani Mat Hassan - International Islamic University Malaysia

Dr. Razul Md Nazri Bin Md Kassim - Hospital Sultahan Bahiyah

Dr. Wong Jyi Lin - Hospital Umum Sarawak

Dr. Fuad Ismail - Hospital Universiti Kebangsaan

Germany:

Dr. Stephan von Haehling – Charité Medical School, Berlin

1. Stewart Coats AJ, Srinivasan V, Surendran J, Chiramana H, Vangipuram SR, Bhatt NN, Jain M, Shah S, Ali IA, Fuang HG, Hassan MZ, Beadle J, Tilson J, Kirwan BA, Anker SD; on behalf of the ACT-ONE Trial Investigators. The ACT-ONE trial, a multicentre, randomised, double-blind, placebo-controlled, dose-finding study of the anabolic/catabolic transforming agent, MT-102 in subjects with cachexia related to stage III and IV non-small cell lung cancer and colorectal cancer: study design. J Cachexia Sarcopenia Muscle. 2011 Dec;2(4):201-207. Epub 2011 Oct 16. [↑](#endnote-ref-1)
2. Hendrik J. Agteresch, Pieter C. Dagnelie, Ate van der Gaast, Theo Stijnen, and J. H. Paul Wilson. Randomised Clinical Trial of Adenosine 5′-Triphosphate in Patients With Advanced Non-Small-Cell Lung Cancer JNCI J Natl Cancer Inst (2000) 92 (4): 321-328 doi:10.1093/jnci/92.4.321 [↑](#endnote-ref-2)
3. Richard G. Tozer, Patricia Tai, Wilma Falconer, Thierry Ducruet, Annie Karabadjian, Gustavo Bounous, John H. Molson, and Wulf Dröge. Antioxidants & Redox Signaling. February 2008, 10(2): 395-402. doi:10.1089/ars.2007.1919. [↑](#endnote-ref-3)
4. Jatoi A, Dakhil SR, Nguyen PL, Sloan JA, Kugler JW, Rowland Jr KM, et al. A placebo-controlled double blind trial of etanercept for the cancer anorexia/weight loss syndrome: results from N00C1 from the North Central Cancer Treatment Group. Cancer. 2007;110:1396–403. [↑](#endnote-ref-4)
5. Mallinckrodt CH, Clark WS, David SR. Accounting for dropout bias using mixed-effects models. Journal of Biophgroupgroupaceutical Statistics 2001; 11: 9–21. [↑](#endnote-ref-5)
